# Chemical manipulation of m^1^A mediates its detection in human tRNA

**DOI:** 10.1261/rna.079966.124

**Published:** 2024-05

**Authors:** Kinga Pajdzik, Ruitu Lyu, Xiaoyang Dou, Chang Ye, Li-Sheng Zhang, Qing Dai, Chuan He

**Affiliations:** 1 Department of Chemistry, The University of Chicago, Chicago, Illinois 60637, USA; 2 Division of Life Science, The Hong Kong University of Science and Technology (HKUST), Kowloon, Hong Kong SAR, China; 3 Howard Hughes Medical Institute, The University of Chicago, Chicago, Illinois 60637, USA; 4 Department of Biochemistry and Molecular Biology, The University of Chicago, Chicago, Illinois 60637, USA; 5 Institute for Biophysical Dynamics, The University of Chicago, Chicago, Illinois 60637, USA

**Keywords:** *N*^1^-methyl adenosine (m^1^A), high-throughput sequencing, RNA modification, mutation rate, readthrough rate, tRNA

## Abstract

*N*^1^-methyl adenosine (m^1^A) is a widespread RNA modification present in tRNA, rRNA, and mRNA. m^1^A modification sites in tRNAs are evolutionarily conserved and its formation on tRNA is catalyzed by methyltransferase TRMT61A and TRMT6 complex. m^1^A promotes translation initiation and elongation. Due to its positive charge under physiological conditions, m^1^A can notably modulate RNA structure. It also blocks Watson–Crick–Franklin base-pairing and causes mutation and truncation during reverse transcription. Several misincorporation-based high-throughput sequencing methods have been developed to sequence m^1^A. In this study, we introduce a reduction-based m^1^A sequencing (red-m^1^A-seq). We report that NaBH_4_ reduction of m^1^A can improve the mutation and readthrough rates using commercially available RT enzymes to give a better positive signature, while alkaline-catalyzed Dimroth rearrangement can efficiently convert m^1^A to m^6^A to provide good controls, allowing the detection of m^1^A with higher sensitivity and accuracy. We applied red-m^1^A-seq to sequence human small RNA, and we not only detected all the previously reported tRNA m^1^A sites, but also new m^1^A sites in mt-tRNA^Asn-GTT^ and 5.8S rRNA.

## INTRODUCTION

So far, there are over 170 identified RNA modifications in all domains of life (Cappannini et al. 2024). RNA modifications regulate RNA processing, localization, stability, and translation (Roundtree et al. 2017). Aberrant RNA modification may lead to tumorigenesis, neurological disorders, and metabolic diseases (Delaunay et al. 2024). tRNA is the most heavily modified RNA species. On average, one in five nucleosides in tRNA bears modification (Zhang et al. 2022). *N*^1^-methyl adenosine (m^1^A) is a modification found at positions 9, 14, 16, 22, 57, and 58 of different tRNAs (Clark et al. 2016; Oerum et al. 2017). m^1^A in tRNA plays a role in tRNA folding and stability (Voigts-Hoffmann et al. 2007; Motorin and Helm 2010), and it promotes translation initiation and elongation (Saikia et al. 2010; Clark et al. 2016; Liu et al. 2016). Recently, m^1^A58 was reported to play a crucial role in T cell expansion (Liu et al. 2022b). Nucleomethylin catalyzes the m^1^A1,322 formation on human 28S rRNA, which affects the local structure of the large ribosomal subunit (Sharma et al. 2018). In humans, the methyltransferase complex TRMT6–TRMT61A catalyzes the formation of m^1^A in cytosolic tRNA and mRNA (Ozanick et al. 2005; Li et al. 2017), whereas TRMT61B and TRMT10C catalyze m^1^A formation in mitochondrial RNA (Chujo and Suzuki 2012; Vilardo et al. 2012). ALKBH1 and ALKBH3 could act as m^1^A demethylases (Li et al. 2016; Liu et al. 2016). Using LC–MS/MS from human poly(A)^+^ RNA, measured m^1^A levels were reported to be ∼0.02%–0.05% of all adenosines (Dominissini et al. 2016; Li et al. 2016).

m^1^A disrupts canonical base-pairing, causing misincorporation or truncation during cDNA synthesis. ARM-seq and DM-tRNA-seq were developed to sequence tRNA and m^1^A methylation in tRNAs. Both methods use a highly processive thermostable group II intron reverse transcriptase (TGIRT) to increase the readthrough of these modifications. Both methods utilize AlkB to demethylate tRNA to construct control libraries, which enable identification of modifications with high accuracy (Cozen et al. 2015; Zheng et al. 2015). Moreover, advances in data analysis have been introduced to DM-tRNA-seq. Clark et al. (2016) combined misincorporation and truncation rates at the modification site, which allows for semiquantitative modification detection. In mim-tRNA-seq and MSR-seq, increased reaction time with TGIRT or SSIV, respectively, resulted in full-length tRNA reads (Behrens et al. 2021; Watkins et al. 2022).

m^1^A in poly(A)^+^ RNA has been profiled using an m^1^A antibody (Dominissini et al. 2016; Li et al. 2016). However, a later study has shown that the m^1^A antibody could bind the m^7^G-cap (Grozhik et al. 2019). In m^1^A-seq-TGIRT and m^1^A-MAP, m^1^A immunoprecipitation has been combined with a TGIRT-based reverse transcription (RT) reaction, allowing for single nucleotide resolution. To filter out false positives, m^1^A-seq-TGIRT and m^1^A-MAP utilize Dimroth rearrangement to convert m^1^A to m^6^A or AlkB treatment, respectively (Li et al. 2017; Safra et al. 2017). Recently, Liu et al. developed an alternative chemical condition to convert m^1^A to m^6^A using 4-nitrothiophenol-mediated Dimroth rearrangement under slightly acidic conditions. This reaction results in much less RNA degradation compared to conventional Dimroth rearrangement under basic conditions (Liu et al. 2022a). Zhao et al. (2018) reported low primer utilization by TGIRT, which may be a possible reason for discrepancy between different studies, especially for low-abundant mRNA. Directed evolution of HIV RT has been used to improve the mutation rate and readthrough rate at m^1^A sites. Evolved RT-1306 enabled more accurate and quantitative detection of m^1^A (Zhou et al. 2019).

**FIGURE 1.**
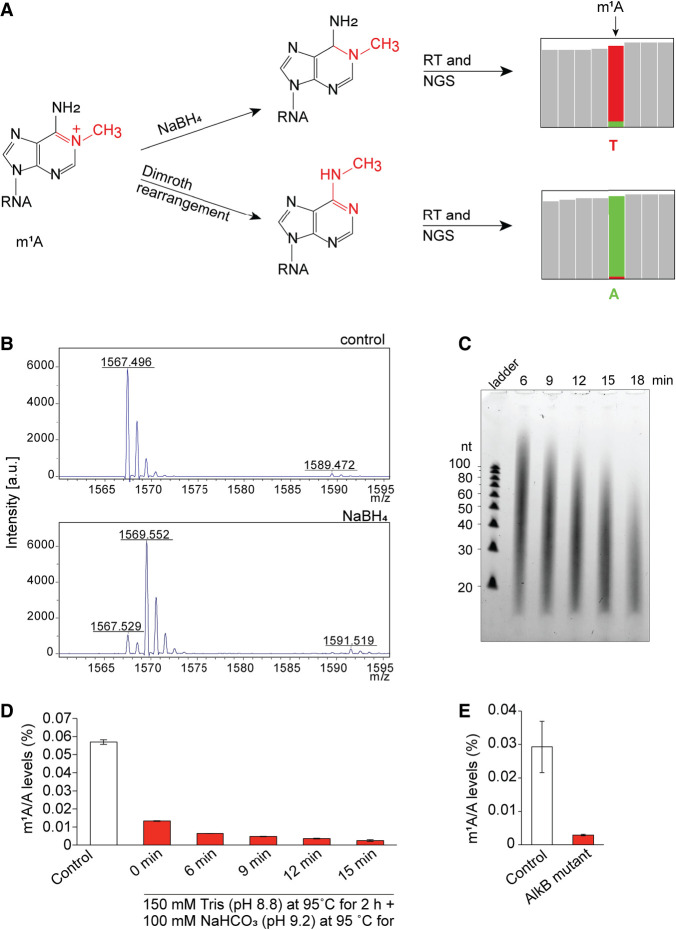
Development of red-m^1^A-seq. (*A*) NaBH_4_ reduction of m^1^A may improve mutation rate and readthrough rate during RT. The modified site could be read as “T” in next-generation sequencing (NGS). Dimroth rearrangement converts m^1^A to m^6^A, and thus m^1^A is read as “A” in NGS. (*B*) MALDI-TOF-MS spectra of m^1^A-containing 5-mer oligonucleotide untreated (control; *m*/*z* = 1567) and treated with 0.1 M NaBH_4_ for 1 h at room temperature. (*C*) TBE-UREA gel of HeLa poly(A)^+^ RNA treated with 150 mM Tris-HCl (pH 8.8) at 95°C for 2 h, followed by an additional treatment with 100 mM NaHCO_3_ (pH 9.2) at 95°C for 6, 9, 12, 15, or 18 min, respectively. (*D*) LC–MS/MS showing m^1^A/A levels in HeLa poly(A)^+^ RNA under different alkaline conditions. HeLa poly(A)^+^ RNA was treated with 150 mM Tris (pH 8.8) at 95°C for 2 h, followed by additional treatment with 100 mM NaHCO_3_ (pH 9.2) at 95°C for 6, 9, 12, or 15 min, respectively. (*E*) LC–MS/MS showing m^1^A/A levels in HeLa poly(A)^+^ RNA upon treatment with AlkB mutant.

Despite these advances in the m^1^A-sequencing protocols, existing methods exhibit some limitations: (i) The number of m^1^A sites with a low modification stoichiometry is likely underestimated due to nonoptimum detection limit (∼8% modification fraction corresponds to a 1% mutation rate) (Zhou et al. 2019); (ii) the calibration curve fits a nonlinear equation, suggesting that RT-1306 may still produce some degree of truncations in biological RNA contexts that decreases sensitivity at certain sites (Zhou et al. 2019); (iii) relying on AlkB demethylation treatment can lead to false negatives, especially for sites that are less abundant or that are located in complex structural contexts inaccessible to enzymatic demethylation treatment; (iv) HIV RT enzyme has low fidelity and thus the background A-to-T mutation rate at unmodified A sites is usually higher than other high fidelity RT enzymes, limiting the sensitivity and accuracy to detect m^1^A sites with low modification level; and (v) both evolved RT enzyme and AlkB are not commercially available, limiting its widespread applications. Since identification of m^1^A sites relies on the mutation rate and its sensitivity to AlkB treatment, seeking an RT enzyme with higher fidelity, RT efficiency and mutation rate at m^1^A sites or AlkB mutant with higher demethylation efficiency should overcome the limitations and further improve the sensitivity and accuracy of the method. Instead of going in this direction, we decided to address these limitations using chemical manipulation of m^1^A by taking advantage of its two unique chemical properties: (i) m^1^A is susceptible to reduction by sodium borohydride (NaBH_4_) (Igo-Kemenes and Zachau 1971; Kubarenko et al. 2001); and (ii) it can be readily converted to m^6^A by Dimroth rearrangement under alkaline conditions (Brookes and Lawley 1960; Macon and Wolfenden 1968). We hypothesized that the reduced m^1^A may generate a higher readthrough rate and a higher A-to-U mutation rate under optimized RT reaction conditions than m^1^A itself due to the loss of the positive charge and the aromaticity of the m^1^A base (Fig. 1A); and chemical conversion of m^1^A to m^6^A by Dimroth rearrangement may be more efficient and robust than the demethylation of m^1^A to A by AlkB treatment, thus providing lower background in the control libraries over AlkB treatment so that comparison of the mutation signatures at the specific sites can improve the m^1^A detection sensitivity and accuracy (Fig. 1A). Here, we describe the development of red-m^1^A-seq, a new m^1^A-sequencing method based on the chemical manipulations of m^1^A in RNA and its application in mapping m^1^A present in human tRNA.

## RESULTS

### m^1^A in RNA can be efficiently reduced by NaBH_4_

First, we optimized the chemical conditions to reduce m^1^A in RNA. We used a synthetic 5-mer RNA oligo containing an m^1^A modification as a model to treat with different concentrations of NaBH_4_ for different time periods, and monitored the reaction by MALDI-TOF-MS. We found that m^1^A was readily reduced within 1 h at room temperature to yield the corresponding product with >80% efficiency (Fig. 1B). When a 33-mer RNA oligo containing an m^1^A was treated with NaBH_4_ for a different length of time, a page gel showed that no obvious RNA cleavage was observed (Supplemental Fig. S1).

### Optimization of Dimroth rearrangement results in efficient m^1^A to m^6^A conversion and suitable RNA fragment size range for library construction

Then we optimized the alkaline conditions under which not only m^1^A is quantitatively converted to m^6^A, but also biological RNA is fragmented to the right size range for subsequent library construction. Our previous experiments showed that alkaline fragmentation of poly(A)^+^ RNA using 0.1 M NaHCO_3_ buffer (pH = 9.2) at 95°C for 5–10 min could degrade RNA to the right size for library construction, but the Dimroth rearrangement efficiency was low. On the other hand, we found that treating RNA with 0.15 M Tris-HCl buffer (pH 8.8) at 95°C for 1 h did not cause serious RNA degradation. Therefore, we decided to incubate poly(A)^+^ RNA with 0.15 M Tris buffer (pH 8.8) at 95°C for 2 h first to promote Dimroth rearrangement, and then add 0.1 M NaHCO_3_ buffer (pH 9.2) and incubate it at 95°C for 15 min to promote the RNA degradation to the right size. As shown in Figure 1C, gel analysis showed that the major fragment size ranges between 40 and 60 bp, which is suitable for the NEBNext Small RNA Library Prep Set for Illumina for library constructions. After digestion to free nucleosides, we used LC–MS/MS to quantify the m^1^A, and we found that the m^1^A/A ratio decreased from 0.055% (untreated) to 0.0023% upon treatment, or a 24-fold reduction (Fig. 1D). In contrast, after the same amount of RNA was fragmented in the presence of Zn^2+^ and then treated with AlkB for demethylation followed by digestion, LC–MS/MS showed that the m^1^A/A ratio was decreased from ∼0.03% to 0.0029%, an ∼10-fold reduction (Fig. 1E). Therefore, our optimized alkaline conditions favor both steps of RNA fragmentation and Dimroth rearrangement.

### Reduction of m^1^A with NaBH_4_ improves RT readthrough rate

**FIGURE 2.**
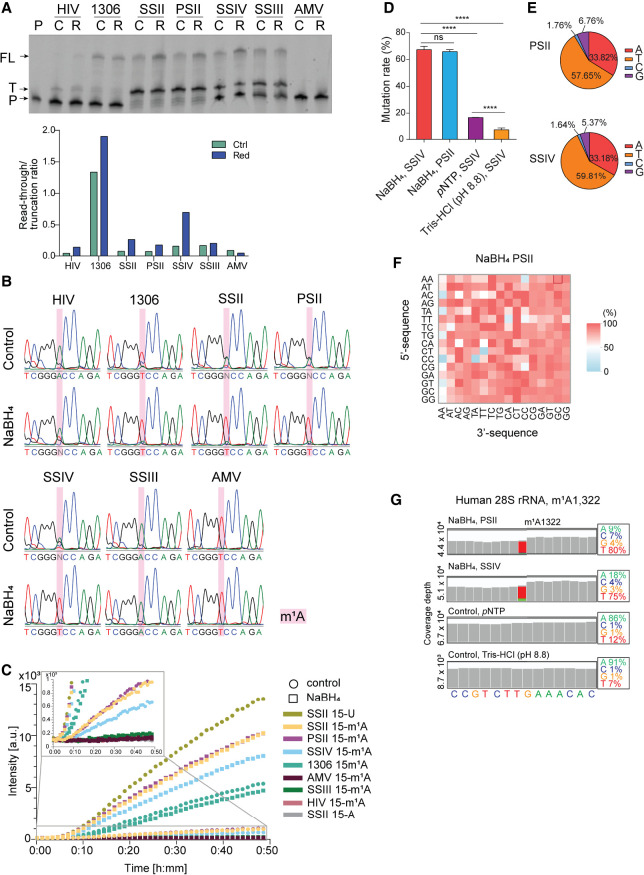
m^1^A reduction with NaBH_4_ improves mutation rate and readthrough rate in RT. (*A*) A gel image of readthrough assay of 33-mer RNA oligonucleotide containing m^1^A without treatment (control, C) or treated with NaBH_4_ (R) using different RT enzymes. (P) Primer, (T) truncated, (FL) full length, (C) control, (R) reduction (*top*). A plot representing quantified readthrough/truncation ratios in readthrough assay. (Ctrl) Control, (Red) reduction (*bottom*). (*B*) Sanger sequencing chromatograms of 33-mer oligonucleotide containing m^1^A. RNA was untreated (control) or treated with NaBH_4_ followed by RT with different RT enzymes followed by PCR and Sanger sequencing. (*C*) Fluorescence intensity measured in the RT-PCR-IVT assay using different RT enzymes with the synthetic 33-mer oligonucleotide containing U, A, or m^1^A at position 15 with or without reduction treatment as the substrate. (*D*) Bar plot showing the mutation rate (%) at the m^1^A modified site in the spike-in RNA oligo with a fixed sequence. These libraries were constructed using the NaBH_4_-, pNTP-, or Tris-HCl (pH 8.8)-treated and SSIV and PSII RT enzymes. (*E*) Pie charts showing the mutation patterns of m^1^A-modified sites in the context of “AAm^1^AGC” within the spike-in RNA oligo as observed in PSII (*top*) and SSIV (*bottom*) RT libraries. (*F*) The heatmap showing the mutation rates at the m^1^A site in the NNm^1^ANN spike-in oligos in the reduction libraries constructed using the PSII RT enzyme. The mutation rates were determined by NGS. Mutation rates in percentage are color-coded. (*G*) Snapshots of IGV coverage tracks showing the mutation signatures of m^1^A1,322 site in human 28S rRNA under different library construction conditions.

To examine the readthrough rate in RT upon m^1^A reduction, we performed a primer extension assay using a 5′-FAM-labeled primer and different RT enzymes (HIV, ProtoScript II [PSII], SuperScript II [SSII], SuperScript III [SSIII], SuperScript IV [SSIV], AMV, HIV, and HIV mutant RT-1306) with NaBH_4_-treated m^1^A-containing 33-mer RNA oligo, with untreated ones as controls. After the RT reaction, we ran a page gel to quantify the unreacted primer, the truncated byproducts, and the desired full-length products (Fig. 2A). The highest RT readthrough/stop ratio was observed for HIV mutant RT-1306. m^1^A reduction with NaBH_4_ further improved the readthrough ratio for RT-1306. The ratio of the full-length/truncated product was higher for the reduced substrate than the untreated one when the WT HIV enzyme was used as well, suggesting that the reduction of m^1^A increases the readthrough rate. The same trend was observed with all other tested RT enzymes except AMV and SSIII. It is also interesting to note that PSII and SSII gave the highest full-length product/unreacted primer ratios, suggesting that they have much higher RT efficiency than HIV and its RT-1306 mutant. While most of the reacted substrate generated truncated product, the formation of a significant amount of the full-length product was also observed, with the reduced m^1^A substrate giving much more full-length RT product than the untreated m^1^A, suggesting that reduction of m^1^A did improve the readthrough rate in general. Despite that the reacted substrate generated RT stop when PSII, SSII, and SSIV were used, the formation of significantly higher amounts of the full-length product was also observed, with the reduced m^1^A substrate affording much more full-length RT product than untreated m^1^A, indicating that the reduction of m^1^A did improve the readthrough rate for commercially available RT enzymes (Fig. 2A).

### Reduction of m^1^A increases misincorporation rate during RT

Next, we set out to compare the mutation rate of the reduced m^1^A substrate with the untreated RNA in an RT reaction using different RT enzymes, followed by PCR and Sanger sequencing. As shown in Figure 2B, the reduced m^1^A substrates gave higher A-to-T mutation rates in all cases, except for AMV and SSIII. We observed that with the untreated substrate, HIV did not introduce misincorporation, while reduction of m^1^A led to ∼30% of A-to-T mutation when HIV was used. Although for RT-1306, the unreduced m^1^A has already shown a good A-to-T mutation rate, the reduced substrate gave an even higher A-to-T mutation rate. Interestingly, while the untreated m^1^A substrate only gave a minimal A-to-T mutation rate when PSII, SSII, or SSIV were used, the reduced substrate gave a nearly quantitative A-to-T mutation, suggesting that the chemical reduction of m^1^A did increase the A-to-T mutation rate in general, and this effect is particularly notable for PSII, SSII, and SSIV.

### Screening RT enzymes to evaluate the combined effect of RT efficiency, readthrough rate, and A-to-T mutation rate

To further evaluate the combined effect of RT efficiency, readthrough rate, and A-to-T mutation rate, we used RT-IVR-PCR assay (Zhou et al. 2019). The 33-mer RNA oligo containing 100% uridine at position 15 served as a positive control, while 33-mer RNA oligo containing 100% adenosine at position 15 served as a negative control. As expected from the readthrough assay and Sanger sequencing results, we observed higher fluorescence intensity for the chemically reduced, m^1^A-containing probe when reversely transcribed with SSII, PSII, SSIV, RT-1306 compared to the unreduced m^1^A-containing probe (Fig. 2C). Interestingly, we did not observe a fluorescence increase for the reduced substrate when using WT HIV (Fig. 2C), even though we observed the A-to-T mutation in Sanger sequencing for the reduced, m^1^A-containing RNA probe. The possible explanation may be the low efficiency of HIV RT, which would require a higher PCR amplification cycle number to produce enough cDNA for in vitro transcription. Although RT-1306 showed a high readthrough rate on the unreduced m^1^A, high A-to-T mutation rate, and therefore a high fluorescence signal, several commercial RT enzymes such as SSII, PSII, and SSIV outperformed RT-1306 when using m^1^A reduction (Fig. 2C). The possible explanation is that RT-1306, similarly to the WT HIV, has relative lower RT efficiency.

### Mutation profile of m^1^A sites detected on the spike-in oligos in NGS

Next, we applied the reduction condition to NGS. Using HepG2 total RNA with synthetic RNA oligos containing m^1^A as spike-in, we built libraries for NGS (Supplemental Fig. S2). As shown in LC–MS/MS (Fig. 1D), m^1^A was still detectable at low levels upon enhanced Dimroth rearrangement conditions. Therefore, we treated RNA for a longer time with Tris-HCl at 95°C for 4, 8, or 16 h to further facilitate m^1^A to m^6^A conversion, followed by additional treatment with 0.1 M NaHCO_3_ at 95°C for 10 min to test whether prolonged treatment could decrease the background (Supplemental Fig. S3A,B). We observed that, for the m^1^A1,322 site in 28S rRNA, treatment for 16 h resulted in the lowest mutation rate (Supplemental Fig. S3A). Therefore, we used this condition for further experiments.

Since PSII showed the highest fluorescence intensity in our RT-PCR-IVT test (Fig. 2C), we used PSII for library preparation. SSIV was successfully used to produce full-length tRNA reads in MSR-seq when RT was prolonged (Watkins et al. 2022). As SSIV also performed well in our studies, we built another set of libraries using SSIV to make comparisons. For both library sets, we prolonged the incubation time with RT enzymes. For the synthetic m^1^A RNA oligo, we observed an ∼65% overall mutation rate at the m^1^A site upon NaBH_4_ reduction when using either PSII or SSIV RT enzymes (Fig. 2D). As shown in Figure 2E, the observed mutation pattern showed that the majority of the mutation originated from A-to-T mutation (57.7% and 59.8% for PSII and SSIV, respectively), and a low rate of A-to-G mutation was observed (6.8% and 5.4% for PSII and SSIV, respectively). The observed mutation rate in the adjacent unmodified adenosines in a synthetic RNA oligo upon treatment with NaBH_4_ was below 1% (Supplemental Fig. S3C).

Besides the enhanced Dimroth rearrangement condition, to make a comparison we also built another set of control libraries, using 4-nitrothiophenol (*p*NTP) (Liu et al. 2022a). In both control libraries, we observed a significant decrease in the mutation rate at the m^1^A site in the synthetic RNA oligo. For *p*NTP-treated control libraries, we observed an ∼16% mutation rate, whereas for Tris-HCl (pH 8.8)-treated, we observed a significantly lower mutation rate, at ∼7% (Fig. 2D), suggesting that our enhanced Dimroth rearrangement condition outperformed the *p*NTP-promoted one.

We then used a synthetic RNA oligonucleotide with an m^1^A site flanked by random sequences (NNm^1^ANN) and tested the mutation rate at the m^1^A site upon reduction with NaBH_4_. We observed consistent mutation rates across all 256 sequence motifs for both PSII (Fig. 2F) and SSIV (Supplemental Fig. S3D, left). For Tris-HCl-treated control libraries, we observed a consistent decrease in the mutation rate (Supplemental Fig. S3D, right).

After demonstrating the proof-of-principle with a synthetic m^1^A RNA oligo, we compared the mutation rate at the well-characterized m^1^A1,322 site in 28S rRNA. The mutation rate at the 28S rRNA m^1^A1,322 in HEK293T reported by m^1^A-quant-seq was 67% for A-to-T mutation, whereas for AlkB-treated libraries, 5% of A-to-T mutation was observed (Zhou et al. 2019). In HepG2, we detected an A-to-T mutation rate as 80% and 75% for PSII and SSIV, respectively. In control libraries, we observed 12% and 7% A-to-T mutation rates upon treatment with *p*NTP and Tris-HCl, respectively (Fig. 2G).

### Application of red-m^1^A-seq to detect m^1^A sites in tRNA

**FIGURE 3.**
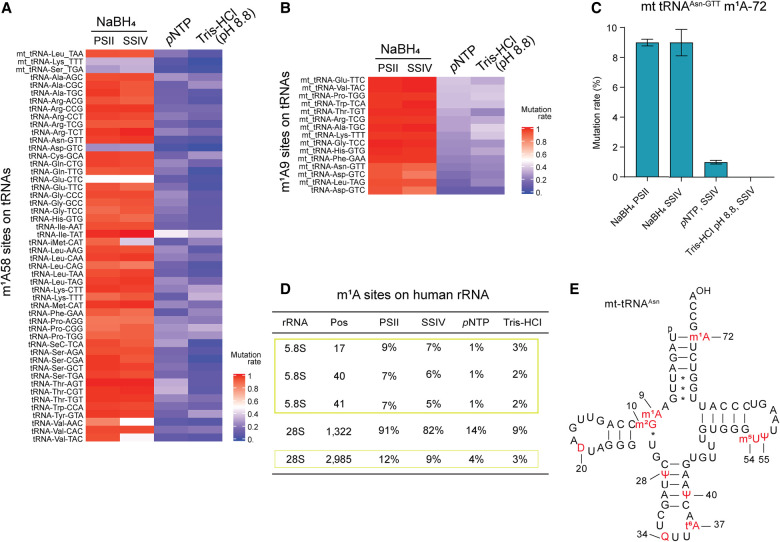
Red-m^1^A-seq detected the known and new m^1^A sites in human tRNA and rRNA. (*A*) Heatmap showing the average mutation rates detected on the m^1^A58 site across 50 tRNA genes using red-m^1^A-seq, based on data from three technical replicates in HepG2 cells. (*B*) Heatmap showing the average mutation rates detected on the m^1^A9 site across 15 tRNA genes using red-m^1^A-seq, based on data from three technical replicates in HepG2 cells. (*C*) Mutation rate at position 72 on mt-tRNA^Asn-GTT^ was measured using red-m^1^A-seq, based on data from three technical replicates in HepG2 cells. Error bars represent SD. (*D*) Table showing the information of m^1^A sites identified in cytosolic rRNA by red-m^1^A-seq. Newly identified m^1^A sites are highlighted in yellow rectangle. (*E*) Secondary structure of mt-tRNA^Asn^ with its reported modifications. (m^2^G) *N*^2^-methylguanosine, (D) dihydrouridine, (m^5^U) 5-methyluridine, (Ψ) pseudouridine, (Q) queuosine, (t^6^A) *N*^6^-threonylcarbamoyladenosine.

After validating the new method with the m^1^A synthetic RNA oligo and known m^1^A1,322 site in 28S rRNA, we then applied red-m^1^A-seq to detect m^1^A sites in human tRNA. We purified HepG2 total RNA and used the RNA Clean & Concentrator Kit (Zymo Research) to isolate the small RNA fraction (<200 nt). Given the size range of tRNA (70–95 nt), we chose not to do further fragmentation so that we could generate longer tRNA reads. m^1^A58 is a well-conserved m^1^A site across different tRNAs. Using our method, we detected robust mutations across 50 cytosolic and mitochondrial tRNAs at position A58 (Fig. 3A). Overall, PSII and SSIV performed similarly in terms of the mutation rate. However, three of the detected sites, tRNA^iMet-CAT^, tRNA^Val-ACC^, and tRNA^Val-TAC^, exhibited significantly lower mutation rates for SSIV compared to PSII (Fig. 3A; Supplemental Table S2). For all the detected m^1^A58 sites, we observed a robust reduction in the mutation rates in both independent control libraries. The average mutation rate for *p*NTP control libraries was 19 ± 8% (mean ± SD), while the average mutation rate for Tris-HCl (pH 8.8) control libraries was 14 ± 9% (mean ± SD) (Fig. 3A). Similarly to m^1^A58, we observed a consistent mutation rate between PSII and SSIV in NaBH_4_-treated libraries at the m^1^A9 site across 15 tRNAs. For PSII, we observed an average mutation rate of 96 ± 3% (mean ± SD), and for SSIV we observed a mutation rate of 95 ± 5% (mean ± SD) (Fig. 3B; Supplemental Table S2). In *p*NTP- and Tris-HCl (pH 8.8)-treated libraries, a significant decrease in mutation rates was observed. The average mutation rate for *p*NTP control libraries was 27 ± 8% (mean ± SD), while the average mutation rate for Tris-HCl (pH 8.8) control libraries was 30 ± 8% (mean ± SD) (Fig. 3B; Supplemental Table S2). Besides these known m^1^A sites, we also identified an m^1^A-72 site in mt-tRNA^Asn-GTT^. We observed a 9% mutation rate for both PSII and SSIV upon NaBH_4_ treatment, but a significant decrease in the mutation rate for controls (1% and 0% in *p*NTP and Tris-HCl [pH 8.8], respectively) (Fig. 3C). To our best knowledge, this site was not reported previously. In addition, we also identified novel m^1^A sites in 5.8S rRNA at positions 17, 40, and 41; as well as in 28S rRNA at position 2985. For all of the new m^1^A sites identified in 5.8 and 28S rRNA, a significant decrease in a mutation rate was observed in *p*NTP and Tris-HCl (pH 8.8) control libraries (Fig. 3D). All novel m^1^A sites in tRNA and rRNA have relatively low modification levels, measured as ≤12% mutation rate. Using m^1^A-red-seq, we report a mutation rate for the well-known m^1^A1,322 site in 28S rRNA as 91% and 82% using PSII and SSIV, respectively (Fig. 3D); these values are consistent with those from previous reports (Clark et al. 2016; Zhou et al. 2019).

## DISCUSSION

Several m^1^A-sequencing methods have been developed so far (Cozen et al. 2015; Zheng et al. 2015; Clark et al. 2016; Dominissini et al. 2016; Li et al. 2016, 2017; Safra et al. 2017; Zhou et al. 2019; Behrens et al. 2021; Watkins et al. 2022). The unique characteristic of m^1^A is the positive charge under physiological conditions and its ability to cause misincorporation during RT. The misincorporation with RT has been utilized in different m^1^A-sequencing protocols (Li et al. 2017; Safra et al. 2017; Zhou et al. 2019). The ability of m^1^A to cause mutation during RT is highly dependent on the reverse transcriptase. In previous studies, TGIRT RT was widely used to map misincorporation caused by m^1^A. However, overlaps of m^1^A sites identified by different studies are rather low (Li et al. 2017; Safra et al. 2017). m^1^A-quant-seq uses evolved RT enzyme with improved readthrough rate and mutation rate at the m^1^A site (Zhou et al. 2019). The control library is necessary to filter out false positives. AlkB demethylase or chemical methods were also used to either demethylate m^1^A or convert it to m^6^A in order to detect real m^1^A sites (Zheng et al. 2015; Li et al. 2017; Safra et al. 2017; Liu et al. 2022a).

In this study, we report that m^1^A reduction with NaBH_4_ improves the readthrough rate and mutation rate at the m^1^A site directly using commercially available RT enzymes, such as PSII or SSIV. Moreover, enhanced Dimroth rearrangement allows for efficient m^1^A to m^6^A conversion, resulting in high-quality control libraries. Although there are some variations probably caused by the sequence context, the general high positive signals in the NaBH_4_-treated libraries and low background in Tris-treated control libraries confirmed that our new method is very effective in m^1^A detection. Liu et al. developed a mild chemical catalysis for m^1^A to m^6^A rearrangement using *p*NTP (Liu et al. 2022a). In this study, we compared both chemical m^1^A to m^6^A rearrangement conditions. Overall, Tris-HCl (pH 8.8)-based m^1^A to m^6^A conversion slightly outperformed *p*NTP. This discrepancy may result from the temperature under which each treatment is performed. *p*NTP treatment is performed at 50°C, whereas Tris-HCl treatment is performed at 95°C. Tris-HCl may result in more efficient m^1^A to m^6^A conversion, as all the secondary structures are diminished at 95°C, and the m^1^A residues are more accessible. However, due to the high temperature of the Tris-HCl (pH 8.8) treatment, *p*NTP may be more applicable for low RNA input samples, for which too much degradation must be avoided.

We applied our method to sequence HepG2 tRNA and detected all known m^1^A-58 sites. One of these sites is m^1^A58 on cytosolic tRNA^Asp-GTC^, which was previously mentioned by Clark et al., but not definitively confirmed due to its very low modification rate (11%) (Clark et al. 2016). Our approach detected this site with a significantly higher m^1^A mutation rate, suggesting that our method may be more applicable to detect lowly modified m^1^A sites. Up to now, over 750 pathogenic sites in mtDNA have been reported, with 300 mutations located within mt-tRNA genes (Brandon et al. 2005). Here, we report a new, lowly modified m^1^A72 site on mt-tRNA^Asn-GTT^ (Fig. 3E). The close proximity of the m^1^A-72 site to the 3′ end of tRNA suggests that this site may play a role in tRNA maturation or tRNA charging. Further studies of the m^1^A-72 biological function and its possible role in disease development are required. Additionally, we detected several new m^1^A sites in rRNA, although all these sites exhibit relatively low modification levels.

Using m^1^A-red-seq, we were able to confirm lowly modified m^1^A58 on cytosolic tRNA^Asp-GTC^, which was previously reported by Clark et al. (2016). Moreover, we reported other lowly modified sites in tRNA and rRNA, which demonstrates the high sensitivity of m^1^A-red-seq. However, m^1^A can spontaneously convert to m^6^A (Suzuki et al. 2020; Ammann et al. 2023). This may affect accuracy, especially for lowly modified sites. Therefore, extra precautions need to be taken during sample handling and storage to avoid underestimation of the m^1^A modification level.

In summary, our method provides a new approach to study m^1^A functions in RNA biology. It uses simple chemical treatment and commercially available RT enzymes and therefore can be widely applied.

## MATERIALS AND METHODS

### DNA and RNA oligonucleotides

RT primer, FAM-labeled RT primer, PCR primers, 3′-RNA adaptor, 3′-cDNA adaptor, and SR RT primer were ordered from Integrated DNA Technologies (IDT), with standard desalting. Other RNA oligonucleotides used in this study were synthesized in-house using an Expedite DNA synthesizer, followed by normal deprotection for regular oligonucleotides and vendor-suggested deprotection for RNA oligonucleotides containing m^1^A modifications to avoid Dimroth rearrangement. After deprotection, the RNA oligonucleotides were purified through HPLC with a C18 column and were eluted with 0%–20% acetonitrile in 0.1 M triethylammonium acetate. The desired peak was collected and dried by SpeedVac Vacuum Concentrator.

### HPLC oligo purification

The deprotected RNA oligo crude mixture was purified by injecting onto a C18 reverse column (Higgins Analytical) using Waters e2695 equipment. The collected peak was dried using a SpeedVac Vacuum Concentrator.

### PAGE gel oligo purification

33-, 43-, and 45-mer RNA oligonucleotides were purified by PAGE gel (15% acrylamide/bis solution 29:1 [Bio-Rad Laboratories], 7 M urea, 1× TBE buffer). One volume of 5–10 μg of RNA crude mixture was mixed with one volume of 2× RNA loading buffer (9 M urea, 100 mM ETDA [pH 8.0], 0.2% [w/v] bromophenol blue), loaded onto the gel and run at 18 W for 1.5–2 h. RNA bands were visualized with a UV lamp and excised from the gel. Gel pieces were crushed with a pipette tip and oligos were eluted by adding 400 μL gel elution buffer (200 mM KCl, 50 mM KOAc, [pH 7.0]). The mixture was incubated overnight at 4°C on a hula mixer. Next, the mixture was spun for 30 min at 4°C at 21,000*g*. Then, the supernatant was mixed with 2.7 volumes of 100% EtOH and precipitated overnight at −80°C. Next, precipitated RNA was spun 21,000*g* at 4°C for 30 min and the RNA pellet was washed twice with prechilled 70% EtOH. The pellet was air dried for 5–10 min at room temperature and then dissolved in 10 mM Tris (pH 7.5).

### NaBH_4_ treatment optimization with MALDI-TOF-MS

Freshly prepared 1 M NaBH_4_ (Sigma-Aldrich 213462) solution in water was added to 5-mer RNA oligo to a final concentration of 10–200 mM and incubated for an hour at rt. After filtration, the reaction mixture was injected onto a C18 reverse column (Higgins Analytical) using Waters e2695 equipment and the desired peaks were collected. One microliter of the collected fraction was mixed with 2 μL matrix (9:1 [vol/vol] ratio of 2′,4′,6′-trihydroxy acetophenone [THAP; 10 mg/mL in 50% acetonitrile and water]:diammonium citrate [50 mg/mL in water]), loaded onto a MALDI plate and MS spectra were collected using Bruker UltrafleXtreme MALDI-TOF-MS in a positive, reflector mode.

### Cell culture

HeLa and HepG2 cells (American Type Culture Collection) were cultured in DMEM medium (Gibco, catalog no. 11995065) supplemented with 10% (v/v) fetal bovine serum (Gibco, catalog no. 16000044), 100 U/mL of penicillin and 100 μg/mL streptomycin (Gibco, catalog no. 15140122) at 37°C with 5% CO_2_.

### RNA extraction

Total RNA from cultured cells was extracted with TRIzol Reagent (Invitrogen, catalog no. 15596026) following the manufacturer's instructions. Poly(A)^+^ RNA was purified using the Dynabeads mRNA DIRECT Purification Kit (Invitrogen, catalog no. 61012) following the manufacturer's instructions. Small RNA fraction was enriched from total RNA using RNA Clean & Concentrator-25 (Zymo, catalog no. R1017).

### AlkB treatment

AlkB treatment was performed as previously described (Zhang et al. 2021). Briefly, 600 ng of poly(A)^+^ RNA was fragmented with RNA Fragmentation Reagent (Invitrogen AM8740) and purified by RNA Clean & Concentrator (Zymo Research). Fifty nanograms was saved as control and the rest was treated in 50-μL reaction volume with the following reagents: 30 mM MES buffer (pH 5.5), 100 µM (NH_4_)_2_Fe(SO_4_)_2_·6H_2_O, 300 µM α-ketoglutarate, 2 mM l-ascorbic acid, 150 mM NaCl, 2 mM MgCl_2_, and 40 µg mL^−1^ BSA with 2.5 µL SUPERase•In RNase Inhibitor and 1.0 µL of engineered AlkB (D135S [Zheng et al. 2015], 10 mg mL^−1^). The mixture was incubated at 25°C for 2 h and then purified using Oligo Clean & Concentrator.

### LC–MS/MS

Two hundred nanograms of RNA was treated with Nuclease P1 (NEB) at 37°C for 2 h followed by rSAP (NEB) treatment at 37°C for 2 h. The reaction mixture was diluted to 60 μL and the samples were filtered through 0.22 μm Millex-GV polyvinylidenedifluoride filters (Millipore). Ten microliters of the solution was injected onto the C18 reverse phase column (Agilent Zorbax Eclipse Plus C18, 2.1 × 50 mm, 1.8 μm) and detected by a triple-quadruple mass spectrometer (Agilent 6495 QQQ) in positive ion multiple reaction monitoring mode. At least three injections were conducted for each sample. The nucleosides were quantified using the nucleoside-to-base ion mass transitions of 268.1–136.2 (A) and 282.1–150.2 (m^1^A). Nucleoside levels were quantified based on standard curves for pure nucleosides.

### Enhanced Dimroth rearrangement and LC–MS/MS

HeLa poly(A)^+^ RNA was mixed with 0.15 M Tris-HCl (pH 8.8) and incubated for 2 h at 95°C. Next, the sample was split into six parts, 0.1 M NaHCO_3_ (pH 9.2) was added, and the sample was treated for an additional 6, 9, 12, 15, or 18 min at 95°C. Samples were then mixed with one volume of 2× RNA loading dye (9 M urea, 100 mM ETDA [pH 8.0], 0.2% [w/v] bromophenol blue) and loaded onto 10% TBE-UREA gel (Invitrogen). ssDNA (IDT) was used as a ladder. After electrophoresis, the gel was incubated with SYBR Gold Nucleic Acid Gel Stain (Invitrogen) and imaged on a gel imager (Bio-Rad).

### Readthrough assay

1.2 μg of 33-mer RNA containing m^1^A was treated in a freshly prepared 0.1 M NaBH_4_ solution in water. The mixture was incubated at room temperature for 1 h in a 1.5 mL Eppendorf tube with a microtube cap lock to prevent tubes from opening due to gas accumulation. After an hour, the tube was opened carefully, and the sample was purified with Oligo Clean & Concentrator (Zymo). RNA was eluted with 12.5 μL water. One hundred nanograms of untreated and 100 ng of treated RNA was used for RT. RNA was first mixed with 5 pmol of FAM RT primer and heated at 65°C for 5 min, then put on ice for at least 2 min before adding RT reaction components. Each RT reaction was set up in 10 μL reaction volume. RT reaction conditions were as follows: (i) 1× First-Strand Buffer (Invitrogen), 1 mM dNTP, 10 mM DTT, 20 U/μL murine RNase inhibitor (NEB M0314), 0.5 μM FAM primer, 10 U/μL SuperScript II (Invitrogen 18064014). Run at 42°C for 1 h followed by 80°C for 10 min. (ii) 1× ProtoScript II buffer (NEB), 1 mM dNTP, 10 mM DTT, 20 U/μL murine RNase inhibitor (NEB M0314), 0.5 μM FAM primer, 10 U/μL ProtoScript II (NEB M0368). Run at 42°C for 1 h followed by 80°C for 10 min. (iii) 1× AMV buffer (NEB), 1 mM dNTP, 10 mM DTT, 20 U/μL murine RNase inhibitor (NEB M0314), 0.5 μM FAM primer, 0.5 U/μL AMV RT (NEB M0277). Run at 42°C for followed by 80°C for 10 min. (iv) 1× First-Strand Buffer (Invitrogen), 1 mM dNTP, 10 mM DTT, 20 U/μL murine RNase inhibitor (NEB M0314), 0.5 μM FAM primer, 10 U/μL SuperScript III (Invitrogen 18080093). Run at 50°C for 50 min followed by 80°C for 10 min. (v) 1× HIV buffer (50 mM Tris-HCl [pH 8.3], 75 mM KCl and 2 mM MgCl_2_), 1 mM dNTP, 10 mM DTT, 20 U/μL murine RNase inhibitor (NEB M0314), 0.5 μM FAM primer, 1 μM HIV WT (Zhou et al. 2019). Run at 37°C for 1 h followed by 80°C for 10 min. (vi) 1× HIV buffer (50 mM Tris-HCl [pH 8.3], 75 mM KCl and 2 mM MgCl_2_), 1 mM dNTP, 10 mM DTT, 20 U/μL murine RNase inhibitor (NEB M0314), 0.5 μM FAM primer, 1 μM RT-1306 (Zhou et al. 2019). Run at 37°C for 1 h followed by 80°C for 10 min. (vii) 1× SSIV buffer (Invitrogen), 1 mM dNTP, 10 mM DTT, 20 U/μL murine RNase inhibitor (NEB M0314), 0.5 μM FAM primer, 10 U/μL SuperScript IV (Invitrogen 18090010). Run at 50°C for 10 min followed by 80°C for 10 min. After the RT reaction, the RT reaction product was mixed with one volume of 2× RNA loading buffer (9 M urea, 100 mM ETDA [pH 8.0], 0.2% [w/v] bromophenol blue), and the RT reaction product was separated on PAGE gel (15% acrylamide/bis solution 29:1 [Bio-Rad Laboratories], 7 M urea, 1× TBE buffer). Bands were visualized on a gel imager (Bio-Rad) using Alexa-488 settings.

### RT-PCR-Sanger sequencing

One hundred and fifty nanograms of 33-mer RNA containing m^1^A was treated in a freshly prepared 0.1 M NaBH_4_ solution in water. The mixture was incubated at room temperature for 1 h in a 1.5 mL Eppendorf tube with a microtube cap lock to prevent tubes from opening due to gas accumulation. After an hour, the tube was opened carefully, and the sample was purified with Oligo Clean & Concentrator (Zymo). RNA was eluted with 31 μL water. Five nanograms of untreated and 5 ng of treated RNA were used for RT. RNA was first mixed with 5 pmol of RT primer and heated at 65°C for 5 min, then put on ice for at least 2 min before adding RT reaction components. Each RT reaction was set up in 10 μL reaction volume. RT reaction conditions were used as described in the “Readthrough assay” section. After RT, 2 μL of RT reaction mixture was used for PCR reaction set up in a total volume of 25 μL. PCR reaction consisted of the following: 1× Standard Taq (Mg-free) Reaction Buffer, 1.5 mM MgCl_2_, 200 μM dNTPs, 200 μM forward/reverse primer, 0.025 U/μL Taq DNA polymerase (NEB M0320). Initial denaturation at 95°C for 30 min. Amplification for 35 cycles: denaturation at 95°C for 20 sec; annealing at 60°C for 30 sec; extension at 68°C for 25 sec. The final extension was run at 68°C for 5 min. The PCR product was then purified by DNA Clean & Concentrator (Zymo) and submitted for Sanger sequencing.

### RT-PCR-IVT

One hundred and fifty nanograms of 33-mer RNA containing m^1^A was treated in a freshly prepared 0.1 M NaBH_4_ solution in water. The mixture was incubated at room temperature for 1 h in a 1.5 mL Eppendorf tube with a microtube cap lock to prevent tubes from opening due to gas accumulation. After an hour, the tube was opened carefully, and the sample was purified with Oligo Clean & Concentrator (Zymo). RNA was eluted with 31 μL water. Five nanograms of untreated and 5 ng of treated RNA was used for RT. RNA was first mixed with 5 pmol of RT primer and heated at 65°C for 5 min, then put on ice for at least 2 min before adding RT reaction components. Each RT reaction was set up in 10 μL reaction volume. RT reaction conditions were used as described in the “Readthrough assay” section. After RT, 1 μL of the RT reaction mixture was used for PCR reaction set up in a total volume of 10 μL. PCR reaction consisted of the following: 1× Standard Taq (Mg-free) Reaction Buffer, 1.5 mM MgCl_2_, 300 μM dNTP, 500 μM forward/reverse primer, 0.15 U/μL Taq DNA polymerase (NEB M0320). Initial denaturation at 95°C for 30 min. Amplification for nine cycles: denaturation at 95°C for 20 sec; annealing at 60°C for 30 sec; extension at 68°C for 25 sec. The final extension was run at 68°C for 5 min. After PCR, 7 μL of a PCR reaction mixture was used for in vitro transcription in a total volume of 21 μL with the following components: 1× T7 polymerase buffer (NEB), 2 mM rNTP mix, 25 mM MgCl_2_, 50 μM DFHBI-1T (LuceRNA), 1.2 U/μL T7 RNA polymerase (NEB M0251). The reaction was run at 37°C and the fluorescence signal was monitored using a plate reader (BioTek) for 1 h with the excitation and emission wavelengths at 472 nm and 507 nm, respectively.

### NGS library preparation

Two micrograms of HepG2 total RNA was fragmented in fragmentation buffer (10 mM ZnCl_2_, 10 mM Tris-HCl [pH 7.5]) at 70°C for 5 min. Immediately after fragmentation, the sample was transferred to ice and EDTA (pH 8.0) was added to the final concentration of 50 mM and fragmented RNA was purified using RNA Clean & Concentrator-5 (Zymo Research). Next, 500 ng of HepG2 small RNA (<200 nt) was mixed with 50 ng of fragmented in the previous step total RNA, 0.5 ng of 43-mer m^1^A RNA oligo and 1 ng of 45-mer m^1^A RNA oligo. 1× T4 PNK buffer (NEB), 0.4 U/μL SUPERase•In (Invitrogen), and 0.8 U/μL T4 PNK (NEB M0201) were added and mixture was incubated at 37°C for 45 min. Next, RNA was purified with RNA Clean & Concentrator-5 (Zymo Research) and eluted with 21 μL water. RNA concentration was measured with Qubit (Invitrogen). RNA volume was then adjusted to 36 μL and split to set up four 3′ RNA ligation reactions. Approximately 100 ng of RNA in 9 μL water was mixed with 1 μL of 11.25 μM 3′ RNA adaptor and incubated at 70°C for 2 min, then kept on ice for 2 min. Next, 2 μL water, 2.5 μL of 10× T4 RNA Ligase Reaction Buffer (NEB B0216S), 7.5 μL of 50% PEG8000 (NEB B1004S), 1 μL of SUPERase•In, and 2 μL of T4 RNA ligase 2 truncated KQ (NEB 0373) were added and the mixture was incubated at 25°C for 1 h followed by incubation at 16°C for 16 h. The next day, to each ligation mixture, 1 μL of 5′ deadenylase (NEB M0331) was added and incubated at 30°C for 1 h. Adaptor excess was digested by adding to each ligation mixture 1 μL of RecJf (NEB M0264) and incubating at 37°C for 1 h. After adaptor digestion, all four ligation mixtures were combined and purified with RNA Clean & Concentrator-5. To further remove the undigested 3′ adaptor, the EtOH volume was adjusted. Briefly, ligation reactions mixture volume was adjusted to 400 μL with water. Then, 400 μL RNA Binding Buffer (Zymo) and 320 μL of 100% EtOH were added to the sample and then loaded onto the column. Further steps were performed according to the manufacturer's instructions. The purified 3′ RNA ligated sample was then divided into three parts: (i) to treat with NaBH_4_, (ii) to treat with *p*NTP, (iii) to treat with Tris-HCl (pH 8.8). Each part was further subdivided into three parts as technical replicates. Treatment was performed as follows: (i) Store 3′ ligated RNA to treat with NaBH_4_ at −80°C until ready to use. (ii) *p*NTP treatment was performed as described previously (Liu et al. 2022a). Briefly, to 3′ ligated RNA in 25 μL water, add 25 μL of 2× reaction mixture (2× stock: 50 mM *p*NTP [Thermo Scientific Chemicals H59240.14], 10 mM THP (Sigma-Aldrich 777854) in 10% (v/v) DMF (Sigma-Aldrich 270547, pH 6). Incubate at 50°C for 16 h. (iii) To 3′ ligated RNA in 40.5 μL water, add 4.5 μL of 1.5 M Tris-HCl (pH 8.8) and incubate at 95°C in a lid heated to 110°C thermoblock for 16 h. Next, add 5 μL of 1 M NaHCO_3_ (pH 9.2) and incubate for an additional 10 min at 95°C. Put on ice immediately and add 10 μL of 3M NaOAc (pH 5.5) buffer to neutralize the sample and prevent from further degradation. Purify *p*NTP- and Tris-treated samples with RNA Clean & Concentrator-5. To remove over-fragmented RNA fragments that could generate too short to map reads, EtOH volume was adjusted. Per 50 μL sample volume. 100 μL RNA binding buffer (Zymo) and 100 μL of 100% EtOH was used. Further steps were performed according to the manufacturer's instructions. Retrieve 3′ ligated RNA from −80°C and perform NaBH_4_ treatment on (i) 3′ ligated RNA, (ii) 3′ ligated and pNTP-treated RNA, (iii) 3′ ligated and Tris-treated RNA as follows: to RNA in 16 μL water, add 2 μL of freshly prepared 1 M NaBH_4_ and 2 μL of 1 M Tris-HCl (pH 7.5). Incubate at room temperature for 1 h in a 1.5 mL Eppendorf tube with a microtube cap lock to prevent tubes from opening due to gas accumulation. After an hour, the tube was opened carefully, and the sample was purified with RNA Clean & Concentrator-5. RT was performed with ProtoScript II (3′ ligated and NaBH_4_-treated RNA only) and SuperScript IV ([i] 3′ ligated and NaBH_4_-treated RNA, [ii] 3′ ligated, *p*NTP- and NaBH_4_-treated RNA, [iii] 3′ ligated, Tris- and NaBH_4_-treated RNA). To RNA in 5 μL water, 1 μL of 5.63 μM SR RT primer was added and incubated at 65°C for 5 min and put on ice for 2 min. For PSII reactions, 4 μL of 5× ProtoScript II buffer, 4 μL water, 2 μL of 10 mM dNTPs, 2 μL of 0.1 M DTT, 1 μL of SUPERase•In, and 1 μL of ProtoScript II (NEB) were added. The reaction was incubated at 42°C for 1 h followed by 16 h incubation at 35°C. For SSIV reactions, 4 μL of 5× SuperScript IV buffer, 4 μL water, 2 μL of 10 mM dNTPs, 2 μL of 0.1 M DTT, 1 μL of SUPERase•In, and 1 μL of SuperScript IV (Invitrogen) were added. The reaction was incubated at 50°C for 1 h followed by 16 h incubation at 35°C. After 16 h incubations, all reactions were treated the same way. RT enzymes were denatured by 10 min incubation at 80°C. Then, 1 μL of RNase H buffer and 1 μL of RNase H (NEB M0297S) were added and the reaction was incubated at 37°C for 30 min. Next, the volume was adjusted to 50 μL with water and samples were purified with RNA Clean & Concentrator-5 (per 50 μL, 100 μL RNA binding buffer and 100 μL of 100% EtOH was added). cDNA was eluted with 7 μL of water and 3′ cDNA ligation was performed as follows: to cDNA in 5–6 μL water, 1 μL of DMSO and 1 μL of 50 μM 3′ cDNA adaptor were added, and incubated at 70°C for 2 min, then put on ice for 2 min. Then, add 2 μL of 10× T4 RNA ligase buffer (NEB), 0.2 μL of 100 mM ATP (NEB), 8 μL of 50% PEG8000 (NEB), 0.6 μL of 50 mM of hexaamminecobalt(III) chloride [Co(NH_3_)_6_Cl_3_] (Sigma-Aldrich 481521), 0.4 μL of 5′ deadenylase (NEB M0331), and 1.5 μL of T4 RNA Ligase 1 (ssRNA Ligase), High Concentration (NEB M0437). Master mix for cDNA ligation was prepared, with RNase Ligase High added last to the master mix and mixed well before adding to the samples. The mixture was incubated at 25°C for 14 h followed by heat inactivation at 65°C for 5 min. The reaction volume was adjusted to 50 μL with water and ligated cDNA was then purified by RNA Clean & Concentrator-5 (per 50 μL sample, 100 μL RNA binding buffer and 80 μL EtOH was added). cDNA was eluted with 21 μL water. LongAmp Taq 2X Master Mix (NEB), SR primer (NEB), and NEBNext Multiplex Oligos for Illumina (NEB) were used for library amplification according to instructions in the NEBNext Small RNA Library Prep Set for Illumina (NEB). Briefly, 10 μL cDNA was used for library amplification in a total volume of 25 μL for 12–13 cycles (NaBH_4_-treated samples), 16 cycles (pNTP-treated cycles), and eight linear cycles (SR primer only) followed by 15 cycles with both PCR primers (Tris-treated samples). Then, 5 μL of 6× Gel Loading Dye, Blue (NEB E6138AA) was added and libraries were separated on 3.5% low melting agarose gel in 1×TBE. Quick-Load pBR322 MspI-DNA Digest (NEB E7323AA) was used as a ladder. Bands corresponding to 180–360 bp were excised from the gel and extracted with MinElute Gel Extraction Kit (Qiagen). Libraries were sequenced on the NextSeq2000 system in a PE63 mode (Illumina).

### Next-generation-sequencing data processing

#### Spike-in oligos sequencing data processing

To ensure the quality and accuracy of our spike-in oligos sequencing data, we implemented a comprehensive preprocessing pipeline. Initially, we used the trim-galore package in paired-end mode to meticulously trim low-quality and adapter-containing reads from the raw sequencing data. We then excluded any reads shorter than 30 bp to maintain a standard of data integrity. In our design, we incorporated “NNNNN” sequences at both ends of the adapter and an additional 6 bp in-line barcode sequence at the 3′ end of the adapter sequence. This allowed us to utilize fastx_collapser from the FASTX-toolkit to efficiently collapse identical sequences in a FASTQ file into a single sequence. This step was crucial for eliminating duplicated reads resulting from PCR amplification. Subsequently, we tailored the collapsed reads by trimming the first 5 bp and the last 5 bp from the R1 reads. Similarly, for the R2 reads, we removed the first 11 bp and the last 5 bp. These modifications were essential for preparing the reads for further analysis. Finally, we utilized the modified reads to calculate mutation rates based on different sequence contexts. 43-mer spike-in oligo with a fixed m^1^A context: 5′-pCCUACCUCCCUCACCAAm^1^AGCCCAUAAAAAU AAAAAAUUAUAAC-3′. 45-mer 43-mer spike-in oligo with a NNm^1^ANN context: 5′-pGUAAUUAUACNNm^1^ANNAUUCGUUGU ACGUGAUGCCUAAUGCCUGAA-3′.

#### Small RNA sequencing data processing

To analyze the small RNA sequencing data from HepG2 cells, we initiated the process by aligning the modified reads to the hg19 reference genome assembly. Raw reverse (R1) reads with depleted PCR duplicates and removed adaptors were mapped to the rRNA (NR_003285.3.fa, NR_003286.4.fa, and NR_003287.4.fa) downloaded from NCBI Nucleotide data, and tRNA (hg19-mature-tRNAs.fa) genomes downloaded from the GtRNAdb database (http://gtrnadb.ucsc.edu/Hsapi19/Hsapi19-seq.html). This alignment was performed using bwa mem under its default parameters. Following this, the mapped SAM files were converted to BAM format and sorted using Samtools sort (v1.9). To refine our data further, we filtered the sorted BAM files using Samtools view (-q 10) to obtain uniquely mapped reads. For mutation identification, we used rnaseqmut utilizing the parameters “-t -s -3 -m 2” to ensure sensitive detection. Our analysis incorporated several stringent cutoffs to filter the mutation list for downstream analysis: (i) We selected mutations with a reference Adenine (A) and a read coverage of at least 50 and a mutation ratio of 5% or greater in both SSIV and PSII libraries, coupled with a *P-*value <0.05. (ii) For two control libraries, we focused on adenine sites with a read coverage of at least 50 and a mutation ratio of <5%. Additionally, we required that mutation ratios in both NaBH_4_ SSIV and PSII libraries be at least twofold higher than those in two control libraries. Additionally, IGV was used for visualizing both spike-in and small RNA-seq data.

### Statistical analysis and visualization using R and GraphPad prism

For the visualization of mutation patterns of m^1^A in the NNm^1^ANN spike-in oligo, we utilized the “ComplexHeatmap” package in R (version 3.6.3). The majority of the bar plots and pie charts were generated with GraphPad Prism 7. To assess the statistical significance of mutation rates at the m^1^A-modified sites in the spike-in oligo with a fixed sequence across different libraries, we conducted a two-tailed Student's *t*-test.

## DATA DEPOSITION

All next-generation-sequencing (NGS) data are available at NCBI Gene Expression Omnibus with the accession number GSE253657. Other data that support the findings of this study are available from the corresponding author upon request.

## SUPPLEMENTAL MATERIAL

Supplemental material is available for this article.
